# Is the age of >65 years a risk factor for endoscopic treatment of primary inguinal hernia? Analysis of 24,571 patients from the Herniamed Registry

**DOI:** 10.1007/s00464-015-4209-7

**Published:** 2015-04-22

**Authors:** F. Mayer, M. Lechner, D. Adolf, D. Öfner, G. Köhler, R. Fortelny, R. Bittner, F. Köckerling

**Affiliations:** Department of Surgery, Paracelsus Medical University, Salzburg, Austria; StatConsult GmbH, Magdeburg, Germany; Department of General and Visceral Surgery, Sisters of Charity Hospital, Linz, Austria; Department of General Surgery, Wilhelminenspital, Vienna, Austria; Hernia Center, Winghofer Medicum, Rottenburg am Neckar, Germany; Department of Surgery and Center of Minimally Invasive Surgery, Academic Teaching Hospital of Charité Medical School, Vivantes Hospital, Neue Bergstraße 6, 13585 Berlin, Germany

**Keywords:** Inguinal hernia, TAPP, TEP, Complications, Age, Reoperation

## Abstract

**Introduction:**

Several analyses of hernia registries have demonstrated that patients older than 65 years have significantly higher perioperative complication rates compared with patients up to the age of 65. To date, no special analyses of endoscopic/laparoscopic inguinal hernia surgery or of the relevant additional influence factors have been carried out. Besides, there is no definition to determine whether 65 years should really be considered to be the age limit.

**Methods:**

In the Herniamed Hernia Registry, it was possible to identify 24,571 patients with a primary inguinal hernia and aged at least 16 years who had been operated on between September 1, 2009, and April 15, 2013, using either the TAPP technique (*n* = 17,214) or TEP technique (*n* = 7,357). Patients in the age group up to and including 65 years (≤65 years) were compared with those older than 65 years (>65 years) in terms of their perioperative outcome. That was done first using unadjusted analysis and then multivariable analysis.

**Results:**

Unadjusted analysis revealed significantly different results for the intraoperative (1.19 vs 1.60 %; *p* = 0,010), postoperative surgical (2.72 vs 4.59 %; *p* < 0.001) and postoperative general complications (0.85 vs 1.98 %; *p* < 0.001) as well as for complication-related reoperations (1.07 vs 1.37 %; *p* = 0,044), which were more favorable in the ≤65 years age group. However, in multivariable analysis, it was not possible to confirm that for the intraoperative complications or the reoperations. Reoperations were needed more often for bilateral procedures (*p* < 0.001; OR 2.154 [1.699; 2.730]), higher ASA classification (IV vs I: *p* = 0.004; OR 6.001 [1.786; 20.167]), larger hernia defect and scrotal hernias. The impact of these factors, in addition to that of age >65 years, was also reflected in the postoperative complication rates. The age limit for increased onset of perioperative complication rates tends to be more than 80 rather than 65 years.

**Conclusion:**

The higher perioperative complication rate associated with endoscopic/laparoscopic inguinal hernia surgery in patients older than 65 years is of multifactorial genesis and is observed in particular as from the age of 80 years.

The demographics of western society are undergoing a significant change, with an increasingly elderly population. Inguinal hernia repair remains the most frequent surgical intervention in the west, and the impact of changing patient demographics means an increasing number of elderly patients require elective surgical repair. Incidence is also higher in the elderly, as loss of tissue strength leads to increased herniation [1–3].

On the basis of the 2006 National Hospital Discharge Survey, patients aged 65 and older accounted for 35 % of all procedures [4]. A nationwide prevalence study showed that the age distribution of inguinal hernia repair is bimodal peaking at early childhood and old age (75–80 years) [5]. The cumulative incidence of inguinal hernia in the USA varies according to the patients’ age: 25- to 39-year-old patients show an incidence of 7.3, 14.8 % at the age of 40–59 years and 22.8 % at the age of more than 60 years [6].

The main goals of elective hernia surgery are symptomatic improvement and prevention of acute surgical emergencies such as incarceration or strangulation. Emergency repair is known to carry significantly higher rates of morbidity and mortality, especially among the elderly [7, 8]. However, there remains a lack of clarity about the appropriateness of intervention in elderly patients with comorbidity in whom symptoms may be minimal and elective repair carries risk [9]. Although a period of watchful waiting has been advocated by some authors for young fit patients, for older patients with comorbidity early elective repair has been advocated [10, 11].

Outcome studies demonstrate that morbidity and mortality are increased following surgery in the elderly as compared with the younger population [12].

In the Swedish Hernia Registry, there was a significant and substantial increase in risk of a postoperative complication with laparoscopic and open preperitoneal procedures in older patient (age > 65 years) [13]. In the Danish Hernia Registry, complications after groin hernia repair were more frequent in patient >65 years (4.5 %), compared with younger patients (2.7 %) (*p* = 0.001) [14].

In the National Surgical Quality Improvement Program (NSQIP) of the American College of Surgeons, the risk of onset of perioperative complications in patients >65 years is expressed with a significant higher odds ratio of 1.418 [1.206–1.666] [15].

Cardiac events occur in 1–5 % of patients undergoing non-cardiac surgery and pulmonary complications in 2.1–10.2 % of elderly patients [4].

The use of a low-pressure pneumoperitoneum and of alternative gases to the CO_2_ pneumoperitoneum is under discussion in order to reduce the cardiopulmonary complications associated with laparoscopic/endoscopic surgery [16, 17]. Other authors exclude older patients with important comorbidities such as severe chronic obstructive pulmonary disease (COPD) from laparoscopic/endoscopic surgical procedures [18].

In the prospective randomized Veterans Affairs Cooperative Study, it was not possible to identify any link between the patient’s age and onset of short-term complications following laparoscopic/endoscopic inguinal hernia surgery (*n* = 989 patients) [19]. In a retrospective comparative study with 185 patients, no difference was discerned in the perioperative complication rates between patients >65 and ≤65 years [18].

In retrospective comparative studies with 104 and 81 patients, no significant difference was seen in the perioperative outcome of patients aged ≥80 years between open and laparoscopic/endoscopic inguinal hernia repair [20, 21].

In an effort to minimize elective operative morbidity and enhance postoperative recovery, laparoscopic repair has been suggested as an appropriate technique to inguinal hernia repair [8, 22, 23].

Existing guidelines do not make any age-specific recommendations on optimal surgical approach in inguinal hernia surgery. There remains a lack of clarity about the safety of laparoscopic repair in an aging population [20, 21].

This study therefore aimed to clarify the impact of age on postoperative outcome after endoscopic repair of primary inguinal hernia, as well as attempting to identify other influence factors that impacted the perioperative outcome and a cutoff age at which laparoscopic repair should no longer be advocated.

## Patients and methods

Herniamed is a multicenter, internet-based hernia registry [24] in which 358 participating clinics and surgeons in private practice from Germany, Austria and Switzerland (Status: April 15, 2013) have prospectively registered their patients who had undergone hernia operations [14]. This present analysis now examines the prospective data of all patients who had undergone laparoscopic/endoscopic inguinal hernia repair in transabdominal preperitoneal patch plasty (TAPP) or total extraperitoneal patch plasty (TEP) between September 1, 2009, and April 15, 2013. The inclusion criteria were a minimum age of 16 years, primary inguinal hernia and uni- or bilateral operation. In total, 24,571 patients were enrolled (Table 1). These comprised 17,214 patients aged ≤65 years (70.1 %) and 7357 patients aged >65 years (29.9 %) (Table 1).

**Table 1:** Classification of patients into age groups

Age	*n*	%
≤65 years	17,214	70.06
>65 years	7357	29.94
Overall	24,571	100.00

The groups were formed by dichotomizing the continuous variable ‘age’ into ‘≤65 years’ and ‘>65 years.’ In addition, the relationship of age to the categories of variables of interest is presented and discussed.

The demographic and surgery-related parameters included sex (m/f), ASA classification (I–IV), risk factors, previous operations and hernia defect sizes based on EHS classification (grade I–III), proportion of scrotal hernias, type of anesthesia, elective or emergency and inpatient vs outpatient treatment. The outcome variables defined were the intra- and postoperative as well as general complication rates, reoperation rate, duration of operation and length of hospital stay. Categorical data are presented as absolute and relative frequencies; continuous variables are displayed as mean, median, standard deviation, quantiles and ranges. In the case of skewed distributions as seen for durations, data were log-transformed. For the bilateral patient group, data on the variables given for both sides operated on were aggregated. For inguinal hernia defects of different sizes, the side with the larger defect is given. Classification as scrotal hernia was based on the presence of at least one scrotal hernia for bilateral inguinal hernia. Intra- and postoperative complications were recorded if a complication presented on at least one side. The same method was used to present details of any reoperation.

All analyses were performed with the software SAS 9.2 (SAS Institute Inc. Cary, NY, USA) and deliberately reviewed to the full level of significance. Each *p* value ≤0.05 thus represents a statistically significant result.

After investigating dependency of outcome variables (intra- and postoperative as well as general complications, reoperation rate, duration of operation and length of postoperative hospital stay) on individual factors (age and other characteristics of patients as well as operation) in unadjusted, univariable analyses (Chi-square test, *t* test), multivariable models (continuously scaled outcome: general linear models, binary-scaled outcome: generalized linear models with logit link function) were applied, thus making it possible to analyze the influence of age adjusted by other possible influencing variables. The parameters of models and their corresponding 95 % confidence intervals are reported as results—odds ratios in case of logistic regression and beta estimates in case of general linear models.

The validity of the logistic models was investigated by means of LOESS regression allowing visualization of the relationship between influencing variable and outcome. Only if a monotone increasing or decreasing relationship is seen is the model valid.

## Results

### Unadjusted results

Unadjusted analyses of the influence exerted by patient classification into age groups on patient characteristic variables (Table 2) showed that the patients in the two age groups differed significantly from each other with regard to the majority of the variables analyzed. For example, the proportion of women in the age group over 65 years was significantly greater (*p* < 0.001). Likewise, in that age group, there were significantly more higher ASA classifications, larger hernia defects and more emergency operations (in each case *p* < 0.001). However, it must be borne in mind that due to the large number of cases even small, possibly clinically irrelevant, differences are identified as being significant.

**Table 2:** Demographic data

	≤65 years		>65 years		^*p*^
	*n*	%	*n*	%	
Sex					
Male	15,481	89.93	6351	86.33	<0.001
Female	1733	10.07	1006	13.67	
ASA score					
I	7578	44.02	797	10.83	<0.001
II	8659	50.30	4523	61.48	
III	968	5.62	1997	27.14	
IV	9	0.05	40	0.54	
Defect size (EHS)					
I (<1.5 cm)	3300	19.17	711	9.66	<0.001
II (1.5–3 cm)	10,883	63.22	4396	59.75	
III (>3 cm)	3031	17.61	2250	30.58	
Scrotal hernia (EHS)					
No	16,851	97.89	7110	96.64	<0.001
Yes	363	2.11	247	3.36	
Anesthesia					
Local	14	0.08	6	0.08	1.000
Spinal	34	0.20	14	0.19	
General	17,166	99.72	7337	99.73	
Inpatient/outpatient					
Outpatient	1020	5.93	194	2.64	<0.001
Inpatient	16,194	94.07	7163	97.36	
Degree of urgency					
Elective	17,061	99.11	7213	98.04	<0.001
Emergency	153	0.89	144	1.96	
Operation technique					
TEP	6636	38.55	2759	37.50	0.122
TAPP	10578	61.45	4598	62.50	
Unilateral/bilateral					
Unilateral	12,276	71.31	5311	72.19	0.165
Bilateral	4938	28.69	2046	27.81	

A pronounced significant difference was found in the risk factors as well as in the rate of previous operations (Table 3). In the age group up to and including 65 years, 21.74 % of patients had at least one risk factor, whereas in the age group older than 65 years that applied for almost one-third of patients (*p* < 0.001). Analysis of individual risk factors revealed that, apart from nicotine abuse, all risk factors were represented more commonly in the higher age group. As expected, that was also the case for the rate of previous operations. At 36.66 %, the rate of previous operations was significantly lower in the younger age group compared with the >65 year olds, where more than one out of every two patients had had at least one previous operation (*p* < 0.001).

The unadjusted tests of the influence of age groups on the outcome parameters (Tables 4, 5) showed a significant difference in all perioperative complication rates, reoperation rate as well as in the duration of operation and the length of postoperative hospital stay.

**Table 3:** Risk factors

	≤65 years		>65 years		*p*
	*n*	%	*n*	%	
*Risk factors*					
Overall					
No	13,471	78.26	4970	67.55	<0.001
Yes	3743	21.74	2387	32.45	
Aortic aneurysm					
No	17,196	99.90	7313	99.40	<0.001
Yes	18	0.10	44	0.60	
Antiplatelet medication					
No	16,732	97.20	6402	87.02	
Yes	482	2.80	955	12.98	<0.001
COPD					
No	16,524	95.99	6784	92.21	
Yes	690	4.01	573	7.79	<0.001
Corticoids					
No	17,104	99.36	7270	98.82	
Yes	110	0.64	87	1.18	<0.001
Diabetes					
No	16,752	97.32	6821	92.71	
Yes	462	2.68	536	7.29	<0.001
Coagulopathy					
No	17,098	99.33	7207	97.96	
Yes	116	0.67	150	2.04	<0.001
Immunosuppression					
No	17,135	99.54	7303	99.27	
Yes	79	0.46	54	0.73	0.010
Anticoagulation therapy					
No	17,140	99.57	7077	96.19	
Yes	74	0.43	280	3.81	<0.001
Smoking					
No	14,836	86.19	6988	94.98	
Yes	2378	13.81	369	5.02	<0.001

**Table 4:** Unadjusted analysis of duration of operation and postoperative hospital stay

	≤65 years			>65 years			*p*
	Mean-STD	Mean	Mean + STD	Mean-STD	MW	Mean + STD	
Duration of operation [min]	33.1	51.0	78.6	33.6	52.2	81.2	<0.001
Post-op. hospital stay [days]	1.0	1.6	2.6	1.1	1.8	3.1	<0.001

**Table 5:** Unadjusted analysis of perioperative complications and reoperations

	≤65 years		>65 years		^*p*^
	*n*	%	*n*	%	
Intraoperative complications					
Overall					
Yes	205	1.19	118	1.60	
No	17,009	98.81	7239	98.40	0.010
Bleeding					
Yes	138	0.80	75	1.02	
No	17,076	99.20	7282	98.98	0.098
Injuries					
Overall					
Yes	116	0.67	65	0.88	
No	17,098	99.33	7292	99.12	0.078
Vascular					
Yes	53	0.31	19	0.26	
No	17,161	99.69	7338	99.74	0.606
Bowel					
Yes	11	0.06	14	0.19	
No	17,203	99.94	7343	99.81	0.007
Bladder					
Yes	16	0.09	13	0.18	
No	17,198	99.91	7344	99.82	0.103
Nerve					
Yes	1	0.01	0	0.00	
No	17,213	99.99	7357	100.0	1.000
Postoperative complications					
Overall					
Yes	468	2.72	338	4.59	
No	16,746	97.28	7019	95.41	<0.001
Bleeding					
Yes	121	0.70	110	1.50	
No	17,093	99.30	7247	98.50	<0.001
Bowel injury/anastomotic leakage					
Yes	6	0.03	3	0.04	
No	17,208	99.97	7354	99.96	0.733
SSI					
Yes	19	0.11	4	0.05	
No	17,195	99.89	7353	99.95	0.255
Seroma					
Yes	319	1.85	224	3.04	
No	16,895	98.15	7133	96.96	<0.001
Mesh infection					
Yes	10	0.06	1	0.01	
No	17,204	99.94	7356	99.99	0.191
Ileus					
Yes	13	0.08	8	0.11	
No	17,201	99.92	7349	99.89	0.475
Reoperation					
Yes	184	1.07	101	1.37	
No	17,030	98.93	7256	98.63	0.044
General complications					
Overall					
Yes	147	0.85	146	1.98	
No	17,067	99.15	7211	98.02	<0.001
Urinary tract infection					
Yes	14	0.08	7	0.10	
No	17,200	99.92	7350	99.90	0.812
Thrombosis					
Yes	4	0.02	3	0.04	
No	17,210	99.98	7354	99.96	0.434
Pulmonary embolism (PAE)					
Yes	2	0.01	3	0.04	
No	17,212	99.99	7354	99.96	0.162
Pneumonia					
Yes	3	0.02	9	0.12	
No	17,211	99.98	7348	99.88	0.002
COPD					
Yes	8	0.05	9	0.12	
No	17,206	99.95	7348	99.88	0.059
Myocardial infarction					
Yes	3	0.02	11	0.15	
No	17211	99.98	7346	99.85	<0.001
Renal failure					
Yes	2	0.01	11	0.15	
No	17212	99.99	7346	99.85	<0.001
Death					
Yes	1	0.01	4	0.05	
No	17213	99.99	7353	99.95	0.031

While the median length of hospital stay (Table 4) in both age groups was 2 days, a significant difference of 0.2 days was identified for the mean value, which was more favorable in the younger age category (*p* < 0.001). The significant difference in the duration of operation, which was on average 1.2 min, was accordingly small.

Overall, there were 0.41 % more intraoperative complications in the >65 years age group (*p* = 0.010), which was largely due a higher rate of intestinal injuries and bleeding (Table 5). The difference in postoperative complications at 2.72 versus 4.59 % was even more pronounced to the disadvantage of the >65 years age group (*p* < 0.001). That was imputable in particular to the higher rate of secondary bleeding and of seromas. There was also a difference of 0.3 % in the reoperation rate, again to the disadvantage of the >65 years age group (*p* = 0.044). There were twice as many general complications in the >65 years age group (0.85 vs 1.98 %, *p* < 0.001). The main complications seen were coronary heart disease, myocardial infarction, renal and cardiac failure, pleural effusion and pneumonia, and these occurred more often in the >65 years age group.

### Multivariable results

Because of the differences in the patient characteristics between the two groups, and in particular due to the potential influence exerted by these variables on the outcome variables, unadjusted analysis of the complication rates with respect to age groups can lead to distortions. The results were verified using multivariable models.

Model fit of intraoperative complications, which reflects the suitability of the influence parameters for explaining the values of the outcome variables, was not significant (*p* = 0.199). Therefore, it was not possible to find any evidence that individual variables had a significant influence on onset of intraoperative complications.

The results of analysis of the postoperative complications are illustrated in Table 6 (model fit: *p* < 0.001). The postoperative complication rate is impacted primarily by an advanced hernia disease and the general condition of the patient. Scrotal EHS classification also resulted in an increased complication risk (OR 2.738 [2.078; 3.609]). Likewise, a larger hernia defect significantly increased the postoperative complication risk (*p* < 0,001; II vs I: OR 1.677 [1.285; 2.187]; III vs I: OR 2.471 [1.855; 3.292]). Equally, the overall complication risk was significantly increased by the use of transabdominal preperitoneal patch plasty (TAPP) (OR 2.461 [2.066; 2.931]). With regard to the postoperative complications, as demonstrated by unadjusted analysis, a significantly lower complication risk was identified in the ≤65 years age group (*p* < 0.001; OR 0.718 [0.612; 0.841]). Low ASA classifications (*p* = 0.037; II vs I: OR 1.161 [0.976; 1.381]; III vs I: OR 1.362 [1.071; 1.732]; IV vs I: OR 2.559 [0.891; 7.346]) and a unilateral operation (*p* = 0.041; OR 1.173 [1.007; 1.366]) also significantly reduced occurrence of a postoperative complication.

**Table 6:** Multivariable analysis of postoperative complications

Parameter	*p* value	Variables	OR	95 % CI	
Operation technique	<0.001	TAPP vs TEP	2.461	2.066	2.931
Scrotal hernia (EHS)	<0.001	Yes vs No	2.738	2.078	3.609
Defect size (EHS)	<0.001	II (1.5–3 cm) vs I (<1.5 cm)	1.677	1.285	2.187
		III (>3 cm) vs I (<1.5 cm)	2.471	1.855	3.292
Age	<0.001	≤65 vs >65 years	0.718	0.612	0.841
ASA score	0.037	II vs I	1.161	0.976	1.381
		III vs I	1.362	1.071	1.732
		IV vs I	2.559	0.891	7.346
Bilateral/unilateral	0.041	Bilateral vs unilateral	1.173	1.007	1.366
Sex	0.304	Male vs female	0.884	0.699	1.118

With a prevalence of 3.28 %, that corresponds to 28 postoperative complications for every 1,000 patients from the ≤65 years age group compared with 38 complications for the >65 years age group.

The results of analysis of the reoperation rate are given in Table 7 (model fit: *p* < 0.001). The reoperation rate was influenced primarily by bilaterality of the inguinal hernia operation (*p* < 0.001). Conduct of a bilateral surgical procedure led to significantly more reoperations (OR 2.154 [1.669; 2.730]). Likewise, high ASA classifications resulted significantly more often in reoperation (*p* = 0.004; II vs I: OR 1.058 [0.799; 1.401]; III vs I: OR 1.581; [1.074; 2.327]; IV vs I: OR 6.001 [1.786; 20.167]). The risk of reoperation also rose for scrotal inguinal hernias (*p* = 0.033; OR 1.807 [1.049; 3.111]). The same applied for a large hernia defect (*p* = 0.043: II vs I: OR 1.208 [0.819; 1.780]; III vs I: OR 1.614 [1.051; 2.478]).

**Table 7:** Multivariable analysis of reoperation

Parameter	*p* value	Variables	OR	95 % CI	
Bilateral/unilateral	<0.001	Bilateral vs unilateral	2.154	1.699	2.730
ASA score	0.004	II vs I	1.058	0.799	1.401
		III vs I	1.581	1.074	2.327
		IV vs I	6.001	1.786	20.167
Scrotal hernia (EHS)	0.033	Yes vs No	1.807	1.049	3.111
Defect size (EHS)	0.043	II (1.5–3 cm) vs I (<1.5 cm)	1.208	0.819	1.780
		III (>3 cm) vs I (<1.5 cm)	1.614	1.051	2.478
Operation technique	0.463	TAPP vs TEP	1.096	0.858	1.399
Age	0.695	≤65 vs >65 years	0.947	0.721	1.244
Sex	0.918	Male vs female	0.979	0.650	1.474

Conversely—and contrary to the findings of unadjusted analysis—the reoperation rate did not differ significantly between the two age groups investigated.

Table 8 gives the results of multivariable analysis of the influences impacting general complications (model fit: *p* < 0.001). The general complications were influenced primarily by ASA status (*p* < 0.001). In particular, ASA classification IV increased the complication risk (IV vs I: OR 6.355 [1.892; 21.345]). Onset of general complications was likewise significantly more common in the >65 years age group than in the ≤65 years age group (*p* < 0.001; OR 0.615 [0.473; 0.800]). The use of the TAPP operation method also favored onset of general complications (*p* = 0.005). The corresponding risk rose for a TAPP operation with an odds ratio of OR 1.432 [1.114; 1.841].

**Table 8:** Multivariable analysis of general complications

Parameter	*p* value	Variables	OR	95 % CI	
ASA score	<0.001	II vs I	1.171	0.855	1.604
		III vs I	3.419	2.381	4.911
		IV vs I	6.355	1.892	21.345
Age	<0.001	≤65 years vs >65 years	0.615	0.473	0.800
Operation technique	0.005	TAPP vs TEP	1.432	1.114	1.841
Bilateral/unilateral	0.225	Bilateral vs unilateral	1.169	0.909	1.504
Defect size (EHS)	0.588	II (1.5–3 cm) vs I (<1.5 cm)	0.842	0.595	1.190
		III (>3 cm) vs I (<1.5 cm)	0.906	0.608	1.352
Scrotal hernia (EHS)	0.665	Yes vs No	1.148	0.614	2.146
Sex	0.999	Male vs female	1.000	0.693	1.443

For an overall general complication rate of 1.19 %, that corresponds to occurrence of a complication in around 10 out of every 1000 patients undergoing surgery from the ≤65 years age group compared with 14 out of every 1000 patients for the >65 years age group.

Model fit of the duration of operation was also highly significant (*p* < 0.001). The highly significant impact of the age groups on the duration of operation, which was demonstrated in unadjusted analysis, could only be confirmed as a trend in the multivariable model (Table 9). In reality, a large hernia defect, the presence of a scrotal hernia, surgery for a male patient, the use of TAPP and bilateral operation (in each case *p* < 0.001) led to significant increase in the duration of operation.

**Table 9:** Multivariable analysis of duration of operation

Parameter	*p* value	Variables	Beta	95 % CI	
Intercept	<0.001		3.717	3.603	3.831
Bilateral	<0.001	Bilateral	0.313	0.301	0.324
Operation technique	<0.001	TAPP	0.089	0.078	0.099
Defect size (EHS)	<0.001	I (<1.5 cm)	−0.113	−0.131	−0.096
Defect size (EHS)	<0.001	II (1.5–3 cm)	−0.097	−0.110	−0.084
Scrotal hernia (EHS)	<0.001	Yes	0.182	0.149	0.216
Sex	<0.001	Male	0.046	0.029	0.062
ASA score		I	0.115	0.002	0.229
ASA score		II	0.124	0.011	0.237
ASA score	0.024	III	0.108	−0.006	0.222
Age	0.072	≤65 Jahre	−0.011	−0.023	0.001

Table 10 shows the results of multivariable analysis of the factors influencing the postoperative length of hospital stay (model fit: *p* < 0.001). The postoperative length of hospital stay was significantly increased in the >65 years age group also, when concurrently looking at the other influencing variables (*p* < 0.001). All other influencing variables also had a significant impact on the length of stay. The length of hospital stay was increased in each case by the use of the TAPP operation method, bilaterality of operation as well as by a scrotal hernia. Besides, the postoperative stay was significantly longer for women than for men.

**Table 10:** Multivariable analysis of hospital stay

Parameter	*p* value	Variables	Beta	95 % CI	
Intercept	<0.001		1.107	0.967	1.246
ASA score	<0.001	I	−0.571	−0.709	−0.432
ASA score	<0.001	II	−0.524	−0.662	−0.386
ASA score	<0.001	III	−0.365	−0.504	−0.226
Bilateral	<0.001	Bilateral	0.111	0.097	0.125
Sex	<0.001	Male	−0.124	−0.145	−0.104
Scrotal hernia (EHS)	<0.001	YES	0.203	0.163	0.243
Age	<0.001	≤65 years	−0.056	−0.071	−0.041
Defect size (EHS)	<0.001	I (<1.5 cm)	0.014	−0.008	0.035
Defect size (EHS)	<0.001	II (1.5–3 cm)	0.039	0.023	0.055
Operation technique	0.020	TAPP	0.015	0.002	0.028

**Fig. 1:**
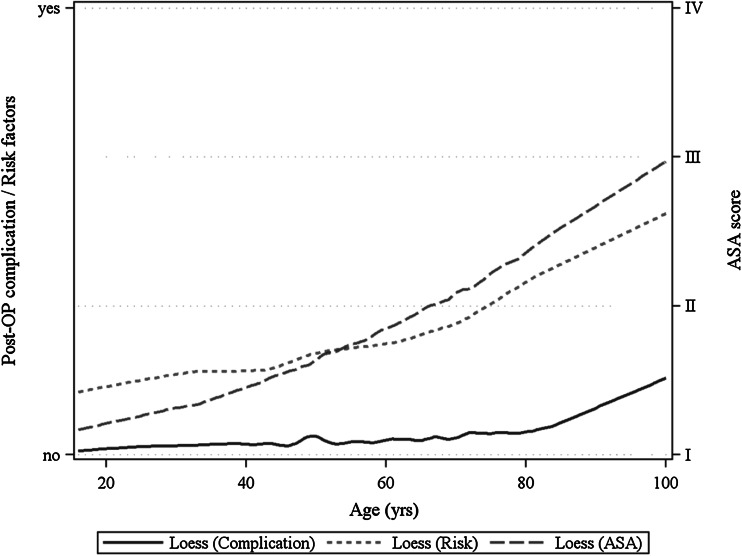
Non-metric regression analysis (LOESS) of age for post-op. complication, cumulative risk factors and ASA score

On the basis of the LOESS graphs, it can be seen that the proportion of higher ASA classifications and of risk factors rise almost linearly with increasing age (Fig. 1). The postoperative complications increase as from age 80 years (Fig. 1).

## Discussion

The registry study presented here investigated the influence of patient age >65 years on the perioperative outcome compared with that of patient age ≤65 years. In addition, other factors impacting onset of perioperative complications were identified and their relative influence on the results determined.

The patients in the two age groups differed significantly from each other with regard to the majority of the variables analyzed. For example, in the >65 years age group, the proportion of women, higher ASA classifications, larger hernia defects, emergency operations, risk factors and previous operations were significantly greater.

Unadjusted analyses revealed that patients in >65 years age group had significantly higher intraoperative, postoperative and general complication rates as well as a higher reoperation rate linked to these complications. In multivariable analysis, it was not possible to find any evidence that individual variables influenced onset of intraoperative complications. As regards the postoperative complications, multivariable analysis showed the risk identified for the ≤65 years age group was significantly lower. Likewise, there were significantly fewer postoperative complications when using TEP, for smaller hernia defects, unilateral operation and a lower ASA classification. Conversely, scrotal EHS classification had an unfavorable influence on occurrence of postoperative complications. However, as in the TAPP group, the percentage of patients with scrotal hernias or with larger hernia defects is significantly higher compared with TEP both minimal invasive techniques are hardly comparable and conclusions should be drawn with caution.

On the other hand, the reoperation rate did not differ significantly between the two age groups in multivariable analysis. The probability of reoperation was increased by bilateral operations, a higher ASA classification, larger hernia defects as well as by a scrotal hernia, but not by the operation technique.

General complications were also seen in multivariable analysis significantly more often in the >65 years age group than in the younger patients. The TAPP operation method and a higher ASA classification are other unfavorable influence factors.

The postoperative length of hospital stay was significantly increased in the >65 years age group, also when concurrently looking at the other influencing variables.

The higher rate of postoperative surgical and general complications in patients in the >65 years age group compared with in the ≤65 years age group thus concords with the findings of the Swedish Hernia Registry [13], Danish Hernia Registry [14] and of the National Surgical Quality Improvement Program of the American College of Surgeons [15]. However, the data presented here in this study additionally show that the age-related rise in postoperative surgical complications did not lead to an increased complication-related reoperation rate. Rather, it was more a bilateral operation, higher ASA classification, larger hernia defect and scrotal hernia which resulted in postoperative complications necessitating reoperation. These were also the influencing variables, in addition to TAPP, which apart from age >65 years gave rise to increased postoperative surgical complications, which could be treated conservatively. Accordingly, the higher perioperative complication rate associated with endoscopic/laparoscopic inguinal hernia surgery in patients >65 years compared with those ≤65 years is of multifactorial genesis. The same applies for the general postoperative complications, which in the >65 years age group were further negatively influenced by conduct of TAPP operation and the presence of a higher ASA classification. That, too, demonstrates the multifactorial influence exerted on the postoperative outcome of patients in the >65 years age group.

The age limit of 65 years is used as a rule for analysis of the influence of age on the postoperative outcome following surgical procedures because of the fact that this is the retirement age in many countries [13]. However, our own analyses based on LOESS graphs show that age 80 years tends to be the time point from which a marked rise is seen in postoperative complications.

In summary, it can be stated that the increase in perioperative complication and reoperation rates associated with laparoscopic/endoscopic inguinal hernia surgery is not only influenced by higher age but mainly by other factors. These include, in particular, bilateral operation, large hernia defect or scrotal hernia and a higher ASA classification and a multitude of risk factors. It can also be demonstrated that it is only as from age 80 years that a relevant rise in postoperative complication rates can be identified. As such, age >65 years in itself does not constitute a risk factor for conduct of laparoscopic/endoscopic inguinal hernia repair. If this is indicated, the focus should be more on the factors identified by the presented study and which have a significant influence on the outcome. In patients over the age of 80, laparoscopic hernia repair is possible, but preoperative analysis of risk factors and their correction if possible should be mandatory [25]. Moreover, careful intraoperative monitoring by the anesthesiologist is essential and the possibility to stay for some hours in an ICU should be provided.

## Appendix: Herniamed Study Group

### Scientific Board

**Köckerling**, Ferdinand (Chairman); **Berger**, Dieter; **Bittner**, Reinhard; **Fortelny**, René; **Koch**, Andreas; **Kraft,** Barbara; **Kuthe**, Andreas; **Lorenz**, Ralph; **Mayer**, Franz; **Moesta**, Kurt Thomas; **Niebuhr**, Henning; **Peiper**, Christian; **Pross**, Matthias; **Reinpold**, Wolfgang; **Simon**, Thomas; **Stechemesser**, Bernd; **Unger**, Solveig

### Participants

**Ahmetov**, Azat (Saint-Petersburg); **Alapatt,** Terence Francis (Frankfurt/Main); **Anders,** Stefan (Berlin); **Anderson**, Jürina (Würzburg); **Arndt**, Anatoli (Elmshorn); **Asperger,** Walter (Halle); **Avram**, Iulian (Saarbrücken); **Barkus**; Jörg (Velbert); **Becker**, Matthias (Freital); **Behrend**, Matthias (Deggendorf); **Beuleke,** Andrea (Burgwedel); **Berger,** Dieter (Baden-Baden); **Bittner,** Reinhard (Rottenburg); **Blumberg,** Claus (Lübeck); **Böckmann,** Ulrich (Papenburg); **Böhle**, Arnd Steffen (Bremen); **Böttger,** Thomas Carsten (Fürth); **Borchert,** Erika (Grevenbroich); **Born**, Henry (Leipzig); **Brabender,** Jan (Köln); **Breitenbuch von**, Philipp (Radebeul); **Brüggemann**, Armin (Kassel); **Brütting**, Alfred (Erlangen); **Budzier**, Eckhard (Meldorf); **Burghardt**, Jens (Rüdersdorf); **Carus**, Thomas (Bremen); **Cejnar**, Stephan-Alexander (München); **Chirikov**, Ruslan (Dorsten); **Comman,** Andreas (Bogen); **Crescent**i, Fabio (Verden/Aller); **Dapunt**, Emanuela (Bruneck); **Decker**, Georg (Berlin); **Demmel**, Michael (Arnsberg); **Descloux,** Alexandre (Baden); **Deusch**, Klaus-Peter (Wiesbaden); **Dick**, Marcus (Neumünster); **Dieterich**, Klaus (Ditzingen); **Dietz**, Harald (Landshut); **Dittmann**, Michael (Northeim); **Dornbusch**, Jan (Herzberg/Elster); **Drummer**, Bernhard (Forchheim); **Eckermann**, Oliver (Luckenwalde); **Eckhoff,** Jörn /Hamburg); **Elger**, Karlheinz (Germersheim); **Engelhardt**, Thomas (Erfurt); **Erichsen,** Axel (Friedrichshafen); **Eucker**, Dietmar (Bruderholz); **Fackeldey**, Volker (Kitzingen); **Farke**, Stefan (Delmenhorst); **Faust**, Hendrik (Emden); **Federmann**, Georg (Seehausen); **Feichter**, Albert (Wien); **Fiedler,** Michael (Eisenberg); **Fischer**, Ines (Wiener Neustadt); **Fortelny**, René H. (Wien); **Franczak**, Andreas (Wien); **Franke**, Claus (Düsseldorf); **Frankenberg von**, Moritz (Salem); **Frehner**, Wolfgang (Ottobeuren); **Friedhoff**, Klaus (Andernach); **Friedrich,** Jürgen (Essen); **Frings**, Wolfram (Bonn); **Fritsche**, Ralf (Darmstadt); **Frommhold,** Klaus (Coesfeld); **Frunder**, Albrecht (Tübingen); **Fuhrer**, Günther (Reutlingen); **Gassler,** Harald (Villach); **Gerdes**, Martin (Ostercappeln); **Gilg**, Kai-Uwe (Hartmannsdorf); **Glaubitz**, Martin (Neumünster); **Glutig,** Holger (Meißen); **Gmeiner**, Dietmar (Bad Dürrnberg); **Göring**, Herbert (München); **Grebe**, Werner (Rheda-Wiedenbrück); **Grothe**, Dirk (Melle); **Gürtler**, Thomas (Zürich); **Hache**, Helmer (Löbau); **Hämmerle**, Alexander (Bad Pyrmont); **Haffner**, Eugen (Hamm); **Hain**, Hans-Jürgen (Groß-Umstadt); **Hammans**, Sebastian (Lingen); **Hampe**, Carsten (Garbsen); **Harrer**, Petra (Starnberg); **Heinzmann**, Bernd (Magdeburg); **Heitland**, Tim (München); **Helbling**, Christian (Rapperswil); **Hempen**, Hans-Günther (Cloppenburg); **Henneking**, Klaus-Wilhelm (Bayreuth); **Hermes**, Wolfgang (Weyhe); **Herrgesell**, Holger (Berlin); **Herzing**, Holger Höchstadt); **Hessler**, Christian (Bingen); **Hildebrand**, Christiaan (Langenfeld); **Höferlin**, Andreas (Mainz); **Hoffmann**, Michael (Kassel; **Hofmann**, Eva M. (Frankfurt/Main); **Hopfe**r, Frank (Eggenfelden); **Hornung**, Frederic (Wolfratshausen); **Hügel**, Omar (Hannover); **Hüttemann**, Martin (Oberhausen); **Huhn**, Ulla (Berlin); **Imdahl**, Andreas (Heidenheim); **Jacob**, Dietmar (Bielefeld); **Jenert**, Burghard (Lichtenstein); **Jugenheimer**, Michael (Herrenberg); **Junger**, Marc (München); **Käs**, Stephan (Weiden); **Kahraman,** Orhan (Hamburg); **Kaiser,** Christian (Westerstede); **Kaiser**, Stefan (Kleinmachnow); **Kapischke**, Matthias (Hamburg); **Karch**, Matthias (Eichstätt); **Keck**, Heinrich (Wolfenbüttel); **Keller,** Hans W. (Bonn); **Kienzle**, Ulrich (Karlsruhe); **Kipfmüller**, Brigitte (Köthen); **Kirsch**, Ulrike (Oranienburg); **Klammer**, Frank (Ahlen); **Klatt**, Richard (Hagen); **Kleemann**, Nils (Perleberg); **Klein**, Karl-Hermann (Burbach); **Kleist**, Sven (Berlin); **Klobusicky**, Pavol (Bad Kissingen); **Kneifel**, Thomas (Datteln); **Knoop**, Michael (Frankfurt/Oder); **Knotter**, Bianca (Mannheim); **Koch,** Andreas (Cottbus); **Köckerling,** Ferdinand (Berlin); **Köhler**, Gernot (Linz); **König**, Oliver (Buchholz); **Kornblum**, Hans (Tübingen); **Krämer**, Dirk (Bad Zwischenahn); **Kraft**, Barbara (Stuttgart); **Kreissl**, Peter (Ebersberg); **Krones**, Carsten Johannes (Aachen); **Kruse,** Christinan (Aschaffenburg); **Kube**, Rainer (Cottbus); **Kühlberg**, Thomas (Berlin); **Kuhn,** Roger (Gifhorn); **Kusch,** Eduard (Gütersloh); **Kuthe,** Andreas (Hannover); **Ladberg**, Ralf (Bremen); **Ladra**, Jürgen (Düren); **Lahr-Eigen**, Rolf (Potsdam); **Lainka**, Martin (Wattenscheid); **Lammers**, Bernhard J. (Neuss); **Lancee**, Steffen (Alsfeld); **Larusson**, Hannes Jon (Pinneberg); **Lauschke**, Holger (Duisburg); **Leher,** Markus (Schärding); **Leidl**, Stefan (Waidhofen/Ybbs); **Lenz**, Stefan (Berlin); **Lesch**, Alexander (Kamp-Lintfort); **Lienert**, Mark (Duisburg); **Limberger**, Andreas (Schrobenhausen); **Locher**, Martin (Kiel); **Loghmanieh**, Siawasch (Viersen); **Lorenz**, Ralph (Berlin); **Mallmann**, Bernhard (Krefeld); **Manger**, Regina (Schwabmünchen); **Maurer**, Stephan (Münster); **Mayer**, Franz (Salzburg); **Menzel**, Ingo (Weimar); **Meurer**, Kirsten (Bochum); **Meyer**, Moritz (Ahaus**)**; **Mirow**, Lutz (Kirchberg); **Mittenzwey**, Hans-Joachim (Berlin); **Mörder-Köttgen**, Anja (Freiburg); **Moesta**, Kurt Thomas (Hannover); Moldenhauer, Ingolf (Braunschweig); **Morkramer**, Rolf (Xanten); **Mosa**, Tawfik (Merseburg); **Müller**, Hannes (Schlanders); **Münzberg**, Gregor (Berlin); **Mussack,** Thomas (St. Gallen); **Neumann**, Jürgen (Haan); **Niebuhr,** Henning (Hamburg); **Nölling**, Anke (Burbach); **Nostitz**, Friedrich Zoltán (Mühlhausen); **Obermaier**, Straubing); **Öz-Schmidt**, Meryem (Hanau); **Oldorf**, Peter (Usingen); **Olivieri**, Manuel (Pforzheim); **Pawelzik**, Marek (Hamburg); **Peiper**, Christian (Hamm); **Pertl**, Alexander (Spittal/Drau); **Philipp**, Mark (Rostock); **Pickart**, Lutz (Bad Langensalza); **Pizzera**, Christian (Graz); **Pöllath**, Martin (Sulzbach-Rosenberg); **Possin**, Ulrich (Laatzen); **Prenzel**, Klaus (Bad Neuenahr-Ahrweiler); **Pröve**, Florian (Goslar); **Pronnet**, Thomas (Fürstenfeldbruck); **Pross**, Matthias (Berlin); **Puff**, Johannes (Dinkelsbühl); **Rabl**, Anton (Passau); **Rapp**, Martin (Neunkirchen); **Reck**, Thomas (Püttlingen); **Reinpold,** Wolfgang (Hamburg); **Reuter**, Christoph (Quakenbrück); **Richter,** Jörg (Winnenden); **Riemann**, Kerstin (Alzenau-Wasserlos); **Rodehorst**, Anette (Otterndorf); **Roehr**, Thomas (Rödental); **Roncossek**, Bremerhaven); **Roth** Hartmut (Nürnberg); **Sardoschau**, Nihad (Saarbrücken); **Sauer**, Gottfried (Rüsselsheim); **Sauer**, Jörg (Arnsberg); **Seekamp**, Axel (Freiburg); **Seelig**, Matthias (Bad Soden); **Seiler**, Christoph Michael (Warendorf); **Seltmann,** Cornelia (Hachenburg); **Senkal,** Metin (Witten); **Shamiyeh**, Andreas (Linz); **Shang**, Edward (München); **Siemssen**, Björn (Berlin); **Sievers,** Dörte (Hamburg); **Silbernik**, Daniel (Bonn); **Simon**, Thomas (Sinsheim); **Sinn**, Daniel (Olpe); **Sinning**, Frank (Nürnberg); **Smaxwil**, Constatin Aurel (Stuttgart); **Schabel**, Volker (Kirchheim/Teck); **Schadd**, Peter (Euskirchen); **Schassen von**, Christian (Hamburg); **Schattenhofer**, Thomas (Vilshofen); **Scheidbach**, Hubert (Neustadt/Saale); **Schelp**, Lothar (Wuppertal); **Scherf**, Alexander (Pforzheim); **Scheyer**, Mathias (Bludenz); **Schimmelpenning,** Hendrik (Neustadt in Holstein); **Schinkel**, Svenja (Kempten); **Schmid**, Michael (Gera); **Schmid,** Thomas (Innsbruck); **Schmidt**, Rainer (Paderborn); **Schmidt**, Sven-Christian (Berlin); **Schmidt,** Ulf (Mechernich); **Schmitz**, Heiner (Jena); **Schmitz**, Ronald (Altenburg); **Schöche**, Jan (Borna); **Schoenen**, Detlef (Schwandorf); **Schrittwieser**, Rudolf /Bruck an der Mur); **Schroll**, Andreas (München); **Schultz**, Christian (Bremen-Lesum); **Schultz**, Harald (Landstuhl); **Schulze**, Frank P. Mülheim an der Ruhr); **Schumacher**, Franz-Josef (Oberhausen); **Schwab**, Robert (Koblenz); **Schwandner**, Thilo (Lich); **Schwarz**, Jochen Günter (Rottenburg); **Schymatzek**, Ulrich (Radevormwald); **Spangenberger**, Wolfgang (Bergisch-Gladbach); **Sperling**, Peter (Montabaur); **Staade**, Katja (Düsseldorf); **Staib**, Ludger (Esslingen); **Stamm**, Ingrid (Heppenheim); **Stark**, Wolfgang (Roth); **Stechemesser,** Bernd (Köln); **Steinhilper**, Uz (München); **Stern**, Oliver (Hamburg); **Stolte**, Thomas (Mannheim); **Stopinski,** Jürgen (Schwalmstadt); **Stubbe**, Hendrik (Güstrow/); **Stülzebach**, Carsten (Friedrichroda); **Tepel,** Jürgen (Osnabrück); **Terzić**, Alexander (Wildeshausen); **Teske,** Ulrich (Essen); **Thews**, Andreas (Schönebeck); **Tillenburg**, Wolfgang (Marktheidenfeld); **Timmermann,** Wolfgang (Hagen); **Train**, Stefan H. (Gronau); **Trauzettel**, Uwe (Plettenberg); **Triechelt**, Uwe (Langenhagen); **Ulcar**, Heimo (Schwarzach im Pongau); **Unger**, Solveig (Chemnitz); **Verweel**, Rainer (Hürth); **Vogel**, Ulrike (Berlin); **Voigt**, Rigo (Altenburg); **Voit**, Gerhard (Fürth); **Volkers**, Hans-Uwe (Norden); **Vossough**, Alexander (Neuss); **Wallasch**, Andreas (Menden); **Wallner**, Axel (Lüdinghausen); **Warscher,** Manfred (Lienz); **Warwas**, Markus (Bonn); **Weber**, Jörg (Köln); **Weiß**, Johannes (Schwetzingen); **Weißenbach**, Peter (Neunkirchen); **Werner**, Uwe (Lübbecke-Rahden); **Wessel,** Ina (Duisburg); **Weyhe**, Dirk (Oldenburg); **Wieber**, Isabell (Köln); **Wiesmann**, Aloys (Rheine); **Wiesner**, Ingo (Halle); **Woehe**, Fritz (Sanderhausen); **Wolf**, Claudio (Neuwied); **Yildirim**, Selcuk (Berlin); **Zarras**, Konstantinos (Düsseldorf); **Zeller**, Johannes (Waldshut-Tiengen); **Zhorzel**, Sven (Agatharied); **Zuz**, Gerhard (Leipzig);
